# Probiotics and Semaglutide Therapy: Mechanistic Perspectives and Potential Interactions in Gut Microbiota, Gastrointestinal Function, and Metabolic Health

**DOI:** 10.3390/nu18213524

**Published:** 2026-10-26

**Authors:** Nazanin Zakeri, Liisa Tolvanen

**Affiliations:** 1 Independent Researcher, Calle Los Angeles, Ur. Polarsol, 29649 Fuengirola, Spain; zakeri.nazanin1@gmail.com; 2 Department of Medicine H7, Karolinska Institutet, 17177 Stockholm, Sweden; 3 Center for Obesity, Academic Specialist Center, Stockholm Health Care Services, P.O. Box 6357, 10235 Stockholm, Sweden

**Keywords:** probiotics, semaglutide, GLP-1 receptor agonists, gut microbiota, gastrointestinal function, gut–brain axis, short fatty acids, intestinal barrier function, metabolic outcomes

## Abstract

Background/Objectives: Semaglutide is one of the currently widely used medications that have shown particularly strong effects on glycemic control and body weight in people with obesity and diabetes. However, gastrointestinal adverse effects are common during semaglutide therapy. Gut microbiota and probiotic supplementation have also attracted growing interest because of their potential effects on metabolic health and gastrointestinal function and their ability to support gastrointestinal health. Methods: This narrative review article investigated the potential interaction between probiotics and semaglutide in relation to gut microbiota, gastrointestinal function, and metabolic health. Results: Patients with metabolic disorders, including type 2 diabetes and obesity, commonly have gut dysbiosis, characterized by an imbalance in the composition and function of the intestinal microbiota. Semaglutide, as a currently important treatment for metabolic disorders, provides effective glycemic control and clinically meaningful weight loss; however, gastrointestinal adverse events are among the most frequently reported adverse effects of semaglutide treatment. Modulation of the gut microbiota may contribute to improved metabolic outcomes and gastrointestinal function. Probiotics may further modulate the gut microbiota by restoring microbial homeostasis, increasing the abundance of beneficial microorganisms, and improving microbial diversity. In addition, probiotic supplementation has been associated with improved metabolic outcomes, including better glycemic control in individuals with diabetes. Conclusions: Probiotics may potentially complement the metabolic effects of semaglutide and support gastrointestinal function and gut microbiota during semaglutide therapy. However, further studies are required to determine whether a clinically meaningful interaction exists between semaglutide and probiotics.

## 1. Introduction

Obesity and type 2 diabetes (T2D) are major public health challenges requiring effective long-term strategies for prevention and treatment [1]. Although lifestyle interventions and pharmacological treatments have advanced considerably, achieving and maintaining clinically meaningful weight loss and metabolic control remains challenging for many individuals [2,3].

Glucagon-like peptide-1 receptor agonists (GLP-1RAs) have expanded substantially in routine clinical practice, particularly in the management of type 2 diabetes and obesity [4,5]. According to a 2025 report from the U.S. National Center for Health Statistics based on the 2024 National Health Interview Survey, 26.5% of adults with diagnosed diabetes reported using injectable GLP-1 medications, corresponding to approximately 6.9 million individuals. Use was higher among individuals with obesity, reaching 32.4% [6]. European registry data also demonstrates a substantial increase in semaglutide use. In Denmark, the prevalence of injectable semaglutide (Ozempic^®^) use increased from approximately 2 per 1000 adults in 2018 to 19 per 1000 in 2023, corresponding to an almost tenfold increase and 91,626 users in 2023 [7]. Similarly, a registry-based study in the Stockholm Region, Sweden, reported that the prevalence of GLP-1 receptor agonist use increased from 4.7 per 1000 inhabitants in 2019 to 17.5 per 1000 in 2023 [8]. These findings illustrate the increasing use of semaglutide and related therapies in European clinical practice and reinforce the importance of understanding treatment tolerability and outcomes.

The clinical efficacy of semaglutide is supported by extensive evidence from the STEP, SUSTAIN, and PIONEER clinical trial programs. However, gastrointestinal adverse events remain one of the most important tolerability considerations. Thus, although semaglutide provides substantial metabolic and cardiovascular benefits, gastrointestinal tolerability remains clinically relevant. This study provides an important context for investigating whether microbiota-targeted strategies, such as strain-specific probiotics, could potentially support gastrointestinal tolerability during semaglutide treatment.

Probiotics are defined as “live microorganisms that, when administered in adequate amounts, confer a health benefit on the “host” [9]. According to the International Scientific Association for Probiotics and Prebiotics (ISAPP), probiotic microorganisms should be adequately characterized, safe for their intended use, and supported by evidence of a health benefit [10]. Importantly, probiotic effects are strain-, dose-, and context-dependent. Selected probiotic strains in this study have been investigated for their potential to improve gastrointestinal symptoms through several mechanisms, including modulation of intestinal motility and bowel habits, reduction of visceral hypersensitivity, support of intestinal barrier integrity, regulation of inflammatory signaling, and production of microbial metabolites. Depending on the strain and clinical context, these effects may be relevant to symptoms such as constipation, diarrhea, abdominal discomfort, and bloating. Whether these effects can improve gastrointestinal tolerability during semaglutide treatment remains uncertain, as direct clinical evidence is currently lacking. Recent reviews have examined the relationship between GLP-1 receptor agonists and the gut microbiota, including potential microbiota-targeted interventions and their metabolic implications. However, whether specific probiotic strains could support gastrointestinal tolerability during semaglutide treatment remains an unresolved clinical question. The present review therefore focuses on strain-specific evidence relating to gastrointestinal symptoms and function, including findings from clinical populations not receiving semaglutide, while distinguishing indirect evidence from direct clinical evidence and identifying priorities for future research. Particular attention is given to the potential relevance of these findings to semaglutide-associated gastrointestinal adverse effects and to the unanswered question of probiotic supplementation timing during different phases of treatment.

## 2. Materials and Methods

We performed a narrative review of the literature focusing on potential probiotic–semaglutide interactions, with a particular emphasis on the gut microbiota, gastrointestinal function, and metabolic health. The literature search was performed mainly in PubMed. Additional relevant studies were searched using Google Scholar. Studies addressing semaglutide and gastrointestinal adverse effects were considered. We used the following search terms, alone and in combination: prevalence of obesity and diabetes, probiotic definition and function, probiotic supplementation and benefits, GLP-1 receptor agonists, semaglutide and obesity, semaglutide and diabetes, semaglutide and cardiovascular outcomes, probiotics and gut microbiota, probiotics and gut–brain axis, semaglutide and gut–brain axis, short-chain fatty acids, semaglutide and gastrointestinal adverse effects, probiotics and intestinal health, semaglutide and metabolic outcomes, probiotics and metabolic health, semaglutide and probiotics, fiber intake and probiotics, and fiber intake and obesity. The literature spanned from 2007 to 2026 and included randomized controlled trials, clinical trials, systematic reviews, meta-analyses, narrative reviews, and relevant mechanistic and experimental studies. Articles were purposively selected based on the relevance to the objectives and thematic focus of the review, especially clinically relevant and recent evidence.

The final narrative synthesis included 109 publications. This study was a narrative review, rather than a systematic review, and therefore no formal systematic review protocol, duplicate independent screening, formal risk of bias assessment, or meta-analysis was performed. The findings were thematically summarized according to the major topics covered in the review: gastrointestinal adverse effects of semaglutide, probiotics and gastrointestinal health, gut microbiota modulation, gut–brain signaling, inflammation and intestinal barrier function, and short-chain fatty acids.

## 3. Results

### 3.1. Semaglutide and Gastrointestinal Adverse Effects

#### 3.1.1. Overview of Semaglutide and Clinical Benefit

**Figure 1:**
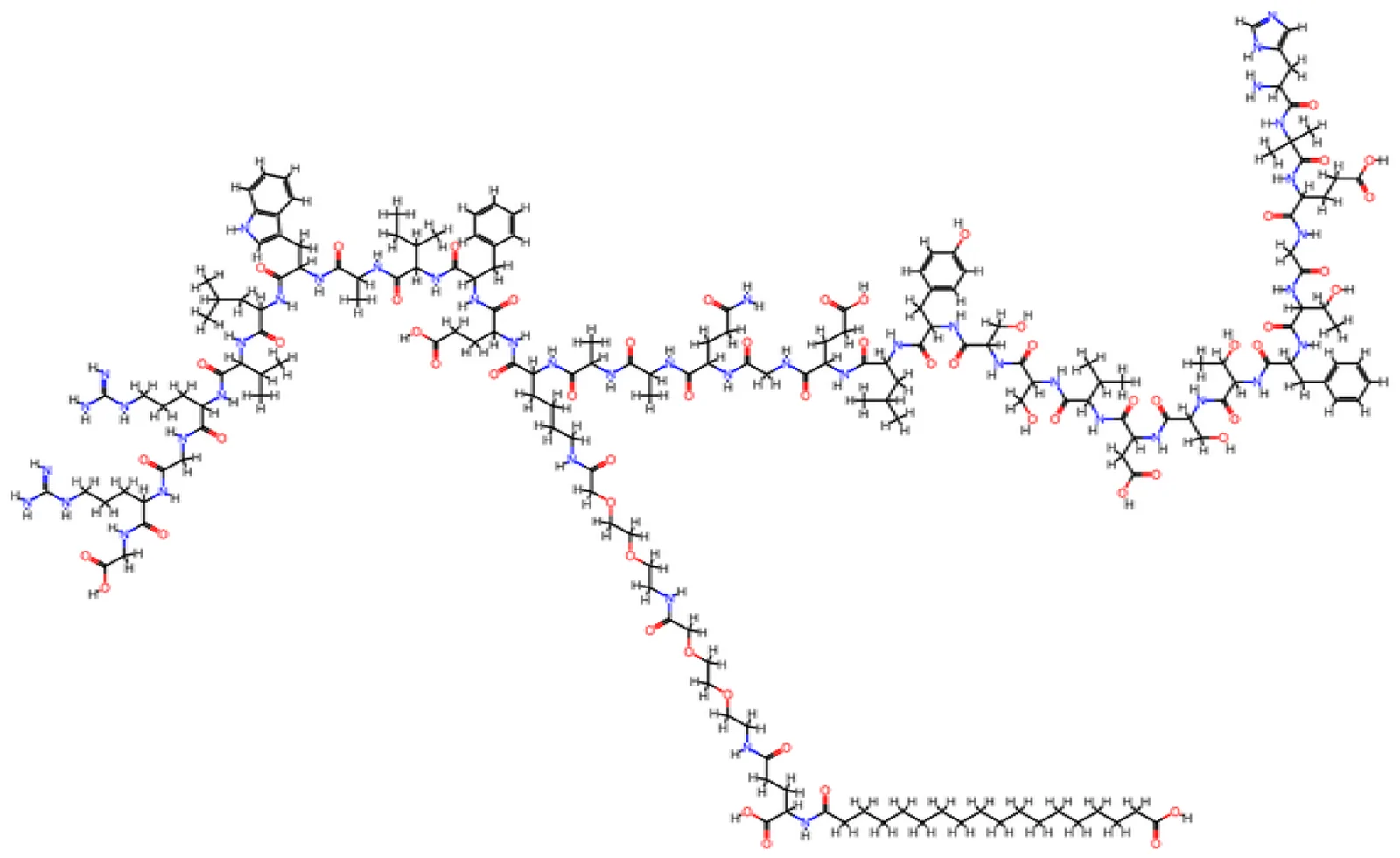
Chemical Structure of Semaglutide. Note. Retrieved from PubChem, National Center for Biotechnology Information (CID 56843331) [22].

Semaglutide is a GLP-1 receptor agonist that is approved for the treatment of obesity and type 2 diabetes and comes in oral and injectable forms [4,5,11]. There is strong clinical evidence for the efficacy of semaglutide in achieving glycemic control with large reductions in HbA1c and clinically meaningful weight loss [12,13,14]. For the chemical structure of semaglutide, see Figure 1. The clinical efficacy of semaglutide is supported by extensive evidence from the STEP, SUSTAIN, and PIONEER clinical trial programs. In the STEP program, once-weekly subcutaneous semaglutide 2.4 mg produced substantial and clinically meaningful weight loss in adults with overweight or obesity, together with improvements in several cardiometabolic risk factors. In STEP 1, participants receiving semaglutide achieved a mean body-weight reduction of 14.9% after 68 weeks, compared with 2.4% with placebo [4]. Importantly, STEP 4 demonstrated that continued treatment was associated with further weight loss, whereas withdrawal of semaglutide resulted in substantial weight regain, highlighting the importance of continued pharmacological treatment for maintaining weight-loss benefits [15]. In individuals with type 2 diabetes, the SUSTAIN program consistently demonstrated improvements in glycemic control and body weight, while SUSTAIN-6 additionally demonstrated a reduction in major adverse cardiovascular events in patients at high cardiovascular risk [16]. The PIONEER program further demonstrated the efficacy of oral semaglutide in reducing HbA1c and body weight in people with type 2 diabetes [17]. Clinical trials on patients with moderate renal impairment showed the efficacy of oral semaglutide, supporting its use in a wide population of T2D patients [18]. Semaglutide is generally well tolerated with a low risk of hypoglycemia. However, gastrointestinal adverse events remain one of the most important tolerability considerations. Across the phase IIIa SUSTAIN and PIONEER programs, gastrointestinal disorders were reported in 41.9% of patients receiving subcutaneous semaglutide and 39.1% of those receiving oral semaglutide, with events occurring most frequently during treatment initiation and dose escalation [19]. Thus, although semaglutide provides substantial metabolic and cardiovascular benefits, gastrointestinal tolerability remains clinically relevant. Most gastrointestinal adverse events are generally mild to moderate in severity and consistent with the GLP-1RA class [4,20,21].

#### 3.1.2. Gastrointestinal Adverse Effects

Gastrointestinal (GI) adverse events are among the most commonly reported adverse effects of semaglutide treatment. In clinical trials, nausea and diarrhea were more common in patients treated with semaglutide than with placebo. In one study, 18% of patients receiving 0.5 mg or 1.0 mg semaglutide reported nausea compared with 7% of patients receiving placebo [23]. Similarly, diarrhea was reported in 13% of patients in both semaglutide groups versus 7% in the placebo group [23].

Comparable outcomes were observed in a separate clinical trial, with nausea reported in 20% of patients taking 0.5 mg semaglutide and 24% of patients taking 1.0 mg, compared to 8% of patients taking placebo [5]. Diarrhea was experienced by 13% and 11% of patients treated with 0.5 mg and 1.0 mg semaglutide, respectively, compared to 2% of placebo [5]. Larger pooled clinical trial data also provide evidence of the gastrointestinal burden associated with semaglutide treatment [24]. In a pooled analysis of the STEP 1–3 trials, the most commonly reported gastrointestinal adverse events with semaglutide 2.4 mg were nausea (43.9%), diarrhea (29.7%), vomiting (24.5%), and constipation (24.2%) versus 16.1%, 15.9%, 6.3%, and 11.1, respectively, in the placebo group [20]. Most gastrointestinal adverse events were mild to moderate, non-serious, transient, and most often during or shortly after dose escalation [20].

Overall, gastrointestinal symptoms, especially nausea, diarrhea, vomiting, and constipation, are important adverse effects of therapy with semaglutide. Such events are typically transient and manageable, but their frequency may influence treatment tolerability and adherence. Thus, understanding the mechanisms of these gastrointestinal effects is important in identifying potential ways to improve gastrointestinal tolerance during semaglutide treatment.

#### 3.1.3. Potential Mechanisms Underlying Gastrointestinal Adverse Effects

Gastrointestinal adverse effects associated with GLP-1 receptor agonists can be broadly classified into upper gastrointestinal symptoms, such as nausea and vomiting, and lower gastrointestinal symptoms, including constipation and diarrhea [25,26]. Upper gastrointestinal symptoms, such as nausea, vomiting, and early satiety, may be associated with delayed gastric emptying and altered central and vagal signaling [26,27].

GLP-1RA can delay gastric emptying [28]. However, this effect can become tachyphylactic rapidly. Repeated administration of GLP-1RA has been shown to significantly decrease the delay in gastric emptying induced by this drug in humans, suggesting that gastrointestinal symptoms observed during long-term therapy are not necessarily due to a persistent delay in gastric emptying [29]. In line with this finding, no evidence of delayed gastric emptying was observed after 20 weeks of treatment in a clinical trial of once-weekly subcutaneous semaglutide 2.4 mg in adults with obesity [30]. However, some studies reported delayed gastric emptying in the early postprandial period [31,32].

Lower gastrointestinal symptoms, including constipation and diarrhea, may involve changes in intestinal motility and gastrointestinal transit time [25,26]. These physiological processes are interconnected rather than entirely independent, as changes in gastric emptying can influence intestinal nutrient delivery, while intestinal feedback mechanisms can, in turn, regulate gastric emptying and gastrointestinal transit [27]. However, the precise contribution of individual pathways to specific symptoms remains incompletely understood [26].

In a recent descriptive case series, a small number of patients receiving GLP-1RA undergoing evaluation for gastrointestinal motility disorders with a wireless motility capsule have been identified with delayed gastric emptying and prolonged whole-gut transit [33]. These findings, however, have limited generalizability due to the small sample size and retrospective single-center design. Thus, alterations in gastrointestinal motility may be an explanation for symptoms such as constipation and abdominal discomfort, but there is inadequate evidence to draw conclusions about a consistent effect of semaglutide on gastrointestinal transit.

**Figure 2:**
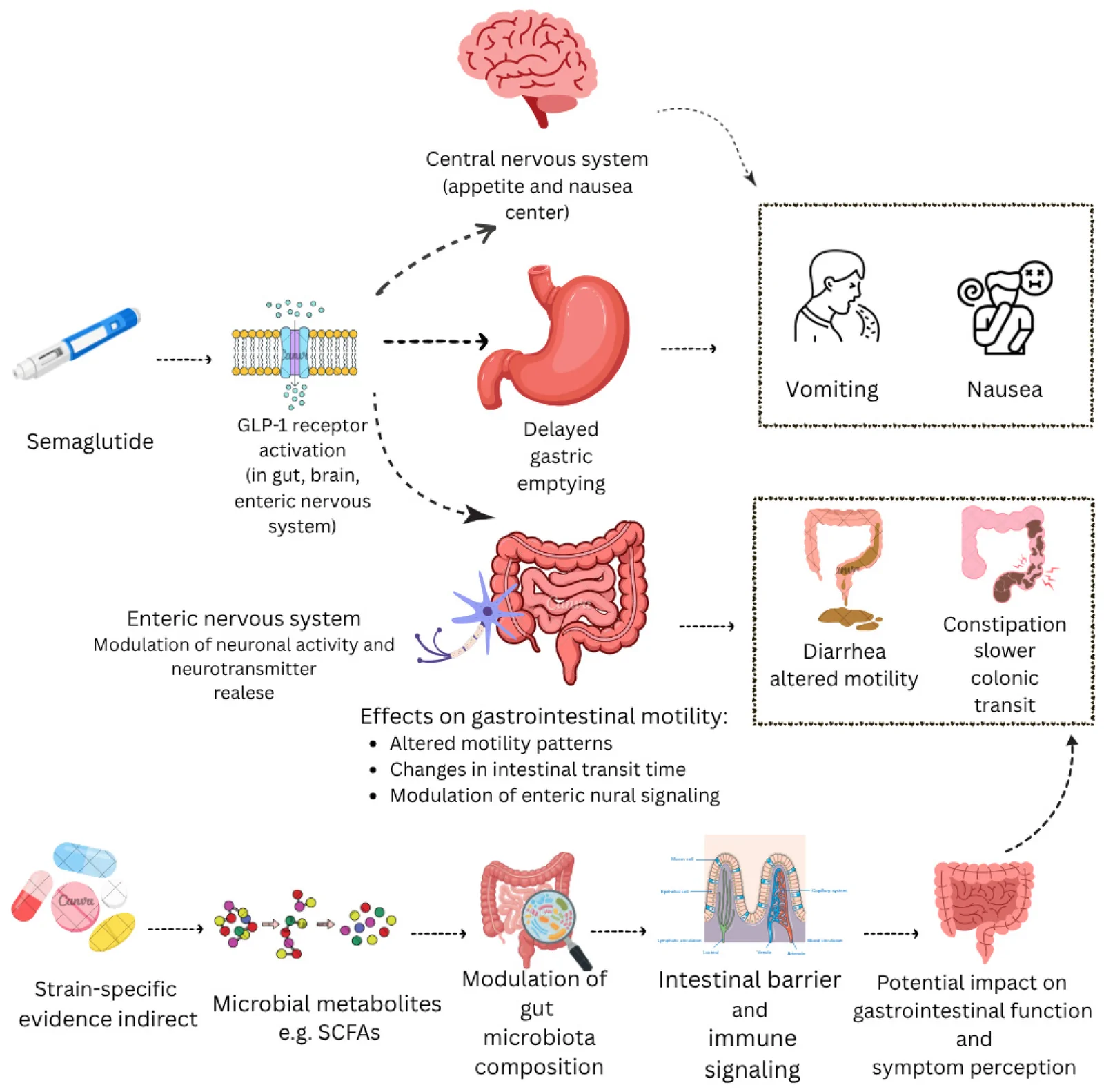
Potential Mechanisms Linking Probiotics and Semaglutide Therapy.

Neural signaling in the enteric nervous system (ENS) may contribute to the gastrointestinal effects of GLP-1 receptor agonists. Endogenous GLP-1 can act on GLP-1 receptors in neural pathways involved in the regulation of gastrointestinal function, although its effects may vary across intestinal regions [34]. Enteroendocrine cells (EECs), which are specialized hormone-producing cells distributed throughout the gastrointestinal epithelium, provide an additional link between the intestinal environment and the nervous system. They sense nutrients and other luminal signals, including signals associated with the gut microbiota, and release mediators such as GLP-1, peptide YY (PYY), cholecystokinin (CCK), and serotonin [35,36]. These mediators can influence enteric neurons and vagal afferent pathways, transmitting information about the intestinal environment to the central nervous system and contributing to the regulation of gastrointestinal motility, secretion, satiety, and visceral sensation [35,36]. In addition, specialized EECs known as neuropod cells can communicate directly with peripheral sensory neurons, providing a rapid route for gut–brain signaling [36]. These pathways are relevant to understanding the potential mechanisms underlying gastrointestinal responses to GLP-1 receptor agonists. Semaglutide activates GLP-1 receptors and affects gastric emptying and gut–brain signaling; however, the extent to which EEC-mediated signaling contributes specifically to semaglutide-associated symptoms, including nausea, bloating, and altered bowel habits, remains incompletely understood; see Figure 2 [27,36]. Taken together, the present data supports that the gastrointestinal effects of semaglutide may be attributed to a combination of changes in gastric emptying, gastrointestinal motility, and enteric neural signaling rather than from a single pathway.

However, further research is needed to better characterize the precise mechanisms underlying upper and lower gastrointestinal symptoms and to determine how patients can best be supported to minimize gastrointestinal adverse effects during semaglutide treatment.

### 3.2. Probiotic Effects on Gastrointestinal Symptoms: Evidence from Other Clinical Contexts

The gut microbiota is a complex microbial community with a major influence on immune regulation, intestinal barrier function, and metabolic health [37]. Disruption in the composition of the gut microbiota, called dysbiosis, has been associated with the development of several metabolic diseases, such as type 2 diabetes, alcohol-associated fatty liver disease, and atherosclerosis [38].

Probiotic supplementation may support microbe homeostasis by regulating the composition of the intestinal microbiota [37]. However, the effects of probiotics on gastrointestinal function are highly strain-specific and may also depend on the dose, duration of administration, formulation, and clinical context. As summarized in Table 1, several individual probiotic strains have been investigated for different gastrointestinal outcomes.

Evidence regarding the effects of probiotics on upper gastrointestinal symptoms, particularly nausea and vomiting, remains limited. In a randomized, placebo-controlled trial, a combination of *Lactobacillus acidophilus* Rosell-52 and *Bifidobacterium longum* Rosell-175 reduced stress-related nausea and vomiting in volunteers [39]. More recently, supplementation with *Bifidobacterium animalis* subsp. *lactis* V9 was associated with improvements in nausea and vomiting scores among patients with advanced gastrointestinal cancer receiving chemotherapy [40]. However, these findings originate from clinical settings unrelated to semaglutide treatment. Therefore, they provide only indirect evidence, and no conclusions can currently be drawn regarding the efficacy of these strains in preventing or alleviating semaglutide-associated nausea and vomiting.

There is some strain-specific evidence regarding the effects of probiotics on lower gastrointestinal symptoms, including bloating, diarrhea, and constipation. *Lactiplantibacillus plantarum* APsulloc 331261 has been evaluated in patients with diarrhea-predominant irritable bowel syndrome (IBS-D), with improvements reported in selected gastrointestinal symptoms [41]. *Bifidobacterium longum* BB536, in combination with *Lactobacillus rhamnosus* HN001, has also been investigated in patients with IBS [42]. Studies of *Lacticaseibacillus rhamnosus* GG and *Lactiplantibacillus plantarum* 299v have reported variable results, highlighting the importance of strain-specific evidence [43,44]. In patients with constipation-predominant IBS, a fermented milk product containing *Bifidobacterium animalis* subsp. *lactis* DN-173 010 has been associated with improvements in selected gastrointestinal outcomes, including abdominal distension and intestinal transit [45]. *Lacticaseibacillus rhamnosus* IDCC 3201 has also been investigated for constipation-related symptoms and bowel satisfaction [46]. While *Bifidobacterium animalis* subsp. *lactis* HN019 has been reported to reduce whole-gut transit time in adults with functional gastrointestinal symptoms [47]. In addition, *Bifidobacterium bifidum* G9-1 has been investigated in patients with type 2 diabetes receiving metformin, although the available exploratory evidence lacks a control group [48]. Conversely, *Bifidobacterium lactis* NCC2818 did not demonstrate significant improvements in gastrointestinal transit or symptoms in patients with chronic constipation [49]. Preclinical evidence has also suggested strain-specific effects on gastrointestinal function. For example, *Bifidobacterium infantis* 35624 reduced visceral pain-related responses in a rat model [50].

Evidence from oncology populations also suggests that the gastrointestinal effects of probiotics may be strain-specific. For example, *Lacticaseibacillus rhamnosus* GG (ATCC 53103) was associated with a lower incidence of severe chemotherapy-related diarrhea and less abdominal discomfort in patients with colorectal cancer [51]. A combination of *Lactobacillus acidophilus* and *Bifidobacterium bifidum* also reduced the incidence of treatment-related diarrhea in patients receiving chemoradiotherapy for cervical cancer [52]. However, these findings were obtained in oncology populations and involved different treatment-related mechanisms and clinical contexts. Therefore, they provide indirect evidence regarding the potential gastrointestinal effects of specific probiotic strains but cannot establish their efficacy in preventing or alleviating semaglutide-associated gastrointestinal adverse effects.

Collectively, these findings illustrate that probiotic effects cannot be generalized across strains, with different preparations showing variable effects on bowel function, transit, visceral sensitivity, and gastrointestinal symptoms. Importantly, these findings were obtained in contexts other than semaglutide treatment. Direct evidence demonstrates that any of these specific strains can prevent or alleviate gastrointestinal adverse effects associated with semaglutide, but it remains lacking. See Table 1.

### 3.3. Potential Interactions Between Probiotics and Semaglutide Therapy

#### 3.3.1. Modulation of Gut Microbiota Composition

Recently the gut microbiota has gained much attention for its contribution to the development of obesity and metabolic diseases. Gut dysbiosis, an imbalance in the composition of the intestinal microbiota, is often associated with metabolic disorders, including type 2 diabetes, obesity, and cardiovascular disease [37]. Consequently, modulation of gut microbiota has been investigated as a potential approach to improving metabolic health [53]. Therefore, the modulation of the gut microbiota through, for example, dietary interventions and fecal microbiota transplantation has emerged as a promising therapeutic target for obesity and associated metabolic disorders [53]. Probiotic supplementation was also associated with improved metabolic outcomes, including glycemic control, in diabetic patients [37].

However, the effects of dietary interventions, including prebiotics, depend partly on the composition and function of the resident microbiota, while synbiotics combine probiotic and prebiotic components and may exert complementary or synergistic effects. Fecal microbiota transplantation involves the transfer of a complex and variable microbial community and raises additional considerations regarding safety and standardization [54,55]. Therefore, probiotics were selected as the primary microbiota-targeted intervention considered in this review because they represent a relatively well-defined and clinically scalable approach in which the strain, dose, and duration of supplementation can be specified.

Probiotics may modulate gut microbiota composition by influencing the abundance and activity of specific microorganisms, but these effects are strain- and context-dependent [56,57]. For example, in a randomized, placebo-controlled trial involving 30 patients with constipation-predominant irritable bowel syndrome (IBS-C), eight weeks of supplementation with *Lacticaseibacillus rhamnosus* IDCC 3201 (RH3201) was associated with increased relative abundances of *Akkermansia muciniphila* and *Bacteroides cellulosilyticus* [46]. However, the study did not involve patients receiving semaglutide, and the clinical relevance of these microbial changes in the context of semaglutide treatment remains unknown.

In a separate preclinical study, combining semaglutide with *A. muciniphila* Akk11 improved selected metabolic, hepatic, and inflammatory outcomes in diabetic mice with MASLD [58]. This study provides experimental evidence of a potential interaction between semaglutide and a specific microbial intervention, but its findings cannot establish clinical efficacy in humans or demonstrate improved gastrointestinal tolerability.

In another preclinical murine study, semaglutide treatment was associated with improvements in intestinal barrier integrity and alterations in the abundance of bacterial genera, including *Alloprevotella* and *Alistipes*, alongside improvements in metabolic dysfunction-associated steatotic liver disease (MASLD) [59]. However, clinical evidence remains limited. In a study of 15 patients with type 2 diabetes inadequately controlled with metformin, 12 weeks of semaglutide treatment was associated with changes in gut microbial composition, including an increased relative abundance of *Bifidobacterium* and a decreased abundance of *Klebsiella*. Although changes in microbial composition were reported, alpha-diversity indices did not change significantly, and the absence of a control group limits causal interpretation [60]. In a separate longitudinal real-world study involving 20 patients with type 2 diabetes, of whom 10 received semaglutide and 10 received empagliflozin, no significant changes in microbial alpha or beta diversity or bacterial genera remained after correction for multiple testing. Nevertheless, baseline microbiome characteristics were associated with subsequent changes in HbA1c, suggesting a possible relationship between the pretreatment microbiome and metabolic response [61]. These findings do not establish a consistent effect of semaglutide on gut microbiota composition and emphasize the necessity of larger controlled studies. The persistence of GLP-1 receptor agonist-associated microbiota changes after treatment discontinuation is also uncertain. In a preclinical study, liraglutide-associated changes in gut bacterial community structure largely reversed after a seven-day washout period [62]. However, these findings were obtained in mice treated with liraglutide and cannot be directly extrapolated to semaglutide-treated humans. Further longitudinal studies are needed to determine whether microbiota changes during GLP-1 receptor agonist treatment persist after treatment cessation and whether they have clinical significance. The available human studies remain limited by small sample sizes, methodological heterogeneity, and the lack of evidence establishing causal relationships between microbiota changes and clinical outcomes. Importantly, changes in microbial composition do not necessarily indicate clinically meaningful benefits. Direct clinical evidence establishing whether probiotics can modify semaglutide-associated microbiota changes or improve treatment outcomes also remains insufficient. See Table 2.

#### 3.3.2. Regulation of the Gut–Brain Axis

The gut–brain axis is a bidirectional communication network that includes neural, hormonal, immune, and metabolic signaling pathways and connects the gastrointestinal tract and central nervous system [69]. Intestinal microbiota and their metabolites can impact brain function directly and indirectly via vagal stimulation or immune-neuroendocrine mechanisms [70,71]. The vagus nerve serves as a major communication route between the gut and the brain and plays an essential role in modulating appetite [72]. Serotonin, one of the key neurotransmitters involved in gut–brain communication that is mainly synthesized in the gastrointestinal tract, activates vagal afferent pathways and modulates intestinal motility and secretion [73].

Experimental evidence indicates that gut microbial activity can influence host serotonin biosynthesis in colonic enterochromaffin cells. Yano et al. demonstrated that selected indigenous spore-forming bacteria and their metabolites promoted serotonin biosynthesis, with consequences for gastrointestinal motility in experimental models [74]. Similarly, Reigstad et al. found that gut microbiota increased colonic expression of TPH1 and serotonin concentrations in mice, while SCFAs promoted TPH1 transcription in a human enterochromaffin-cell model that potentially influenced gut motility and gastrointestinal function [75]. Further evidence suggests that gut microbes may influence host serotonin systems through both direct interactions and indirect mechanisms involving microbial metabolites, including SCFAs, tryptamine, and secondary bile acids [76]. These findings support a potential role for microbiota-mediated serotonin signaling in the regulation of gastrointestinal function, although their relevance to probiotic supplementation and semaglutide-associated gastrointestinal symptoms remain unestablished.

Clinical studies have investigated whether selected probiotic strains can influence gastrointestinal transit, although their findings have been inconsistent. In a randomized, placebo-controlled trial involving 100 adults with functional gastrointestinal symptoms, 14-day supplementation with *Bifidobacterium animalis* subsp. *lactis* HN019 at daily doses of 1.8 × 10^9^ or 17.2 × 10^9^ colony-forming units (CFU) was associated with reductions in whole-gut transit time compared with placebo [47]. In contrast, a subsequent 28-day randomized, placebo-controlled trial involving 228 adults with functional constipation found no significant differences between HN019 and placebo in colonic transit time or the primary and secondary outcomes in the overall study population. A post hoc analysis of 65 participants with fewer than three bowel movements per week suggested an increase in weekly bowel movement frequency with HN019 compared with placebo [77]. However, the result was an exploratory subgroup finding that requires confirmation in further trials. Importantly, neither of these clinical trials directly assessed serotonin biosynthesis, enteroendocrine cell activity, or vagal signaling as mechanisms underlying the observed gastrointestinal outcomes. Thus, although the findings suggest that selected probiotic strains may influence gastrointestinal transit under certain conditions, the mechanisms responsible for these effects remain uncertain. Furthermore, altered gastrointestinal transit alone does not establish that a probiotic modulates the gut–brain axis.

The gut–brain axis is also relevant to the pharmacological actions of semaglutide. GLP-1 receptor agonists influence gastrointestinal function through interconnected central and peripheral pathways, including effects on gastric emptying and neural signaling associated with nausea and vomiting [27]. This provides a biological rationale for investigating whether microbial metabolites or probiotic-associated changes in intestinal signaling could influence gastrointestinal responses during semaglutide treatment. Nevertheless, it remains unknown whether such pathways modify semaglutide-associated gastrointestinal symptoms, metabolic responses, or treatment tolerability.

Overall, the available evidence supports a potential mechanistic connection among gut microbial activity, serotonin signaling, and gastrointestinal function. However, evidence that probiotic supplementation directly modifies gut–brain signaling during semaglutide treatment is currently lacking. These proposed interactions should therefore be regarded as hypotheses rather than established mechanisms of clinical benefit. Further studies should investigate strain-specific effects on microbial metabolites, intestinal signaling, and patient-relevant gastrointestinal outcomes in individuals receiving semaglutide.

#### 3.3.3. Effects of Short-Chain Fatty Acids

The main metabolites resulting from bacterial fermentation of dietary fiber in the gastrointestinal tract are SCFAs, mainly acetate, propionate, and butyrate [78,79]. There is increasing evidence that probiotic microorganisms stimulate the production of SCFAs and maintain a balanced intestinal microbiota. These metabolites are key mediators of host-microbiota interactions and are fundamental in the microbiota-gut–brain axis [80,81]. The biological effects of SCFAs are mediated via various molecular mechanisms. They may activate G-protein-coupled receptors (GPR41 and GPR43) expressed on enteroendocrine L cells to stimulate secretion of endogenous GLP-1, thereby controlling insulin release and appetite [82]. Butyrate is the main energy source for colonocytes and stimulates the formation of epithelial tight junctions; it also improves the integrity of the intestinal barrier and modulates immune response [79]. Emerging evidence suggests that specific gut microbial populations, including *Bacteroides* species, *Akkermansia muciniphila*, and SCFA-producing bacteria, may contribute to GLP-1RA-associated metabolic responses through pathways involving insulin sensitivity, intestinal barrier function, and endogenous GLP-1 secretion [83]. In the context of semaglutide therapy, these microbial pathways could potentially complement pharmacological GLP-1 receptor activation and contribute to metabolic responses through enhanced endogenous GLP-1 signaling and improved insulin sensitivity; see Figure 2 [58,83]. However, the extent to which SCFA-mediated endogenous GLP-1 secretion contributes to the clinical efficacy of semaglutide remains to be fully established. Thus, SCFAs and SCFA-producing microbial populations may represent potential modulators of the gut–metabolic environment during semaglutide treatment, rather than established determinants of treatment efficacy.

#### 3.3.4. Intestinal Barrier Integrity, Inflammatory Responses, and Gastrointestinal Function

Maintaining intestinal barrier integrity is particularly important in obesity and metabolic disorders because the intestinal barrier represents a critical interface between the gut microbiota, the host immune system, and the systemic circulation. The intestinal epithelium, together with the mucus layer, tight-junction proteins, and mucosal immune components, helps regulate the passage of nutrients and microbial products while limiting exposure to potentially inflammatory stimuli. In individuals with obesity, alterations in gut microbial composition and function have been associated with impaired intestinal barrier integrity, including changes in tight-junction proteins and increased intestinal permeability [84]. Increased permeability may facilitate the translocation of lipopolysaccharide (LPS) and other microbial products from the intestinal lumen into the circulation, potentially contributing to metabolic endotoxemia and chronic low-grade inflammation. These inflammatory processes have been implicated in insulin resistance and the development or progression of metabolic complications associated with obesity and type 2 diabetes [85].

However, the extent to which intestinal barrier dysfunction contributes to these conditions varies among individuals, and causal relationships in humans remain incompletely established. Maintaining intestinal barrier integrity and regulating mucosal inflammation may also be relevant to gastrointestinal health. Altered gastrointestinal transit may be associated with changes in gut microbial composition, intestinal barrier function, and mucosal immune responses, particularly in some individuals with chronic constipation [86]. In the context of semaglutide therapy, we hypothesize that treatment-associated changes in gastrointestinal motility or transit could potentially influence gut microbial composition and activity, with possible downstream effects on intestinal barrier integrity and mucosal inflammatory responses. Emerging evidence suggests that probiotic supplementation can influence gut microbial composition and function, with potential implications for metabolic health in individuals with obesity and metabolic disorders [87,88]. Probiotic supplementation may also support gastrointestinal function through modulation of gut microbiota composition, support of intestinal barrier integrity, regulation of immune responses, and production of bioactive metabolites such as short-chain fatty acids [89].

These mechanisms may contribute to the regulation of intestinal inflammation and the maintenance of gut homeostasis, although their effects vary according to the probiotic strain and clinical context. In a randomized, placebo-controlled trial involving adults with obesity, *Bifidobacterium adolescentis* IVS-1 and *Bifidobacterium animalis* subsp. *lactis* BB-12 improved selected measures of intestinal permeability, although the effects differed between strains and no synergistic benefit was observed when probiotics were combined with galactooligosaccharides [90]. In another randomized trial involving adults with excess adiposity, supplementation with *Bifidobacterium bifidum* BGN4 reduced circulating zonulin and TNF-α, whereas the primary inflammatory outcome, hs-CRP, did not change significantly [91]. Importantly, circulating zonulin is an indirect marker that does not independently establish improved intestinal barrier function. Consistent with these limitations, a systematic review of probiotic interventions in individuals with overweight or obesity found heterogeneous results across clinical studies, with inconsistent effects on markers of intestinal permeability [92]. These findings indicate that the effects of probiotics on intestinal barrier-related outcomes are strain-specific and cannot be generalized across interventions.

The effects of semaglutide on gut microbiota composition and intestinal permeability remain incompletely characterized. Preliminary human evidence suggests a possible improvement in intestinal permeability, while small observational studies have reported changes in microbial composition; however, the consistency, causal significance, and clinical relevance of these findings remain uncertain [60,61].

Taken together, direct clinical evidence demonstrates that probiotic supplementation improves intestinal barrier function, reduces inflammation, or enhances gastrointestinal tolerability during semaglutide treatment, but remains lacking. Whether probiotic supplementation can modify semaglutide-associated gastrointestinal adverse effects therefore requires investigation in well-designed, strain-specific clinical trials. See Table 3.

#### 3.3.5. Strain-Specific Effects on Visceral Sensitivity and Intestinal Transit

Strain-specific effects on visceral sensitivity have been investigated in preclinical studies. In a rat study, oral administration of *Bifidobacterium infantis* 35624 for 14 days reduced visceral pain-related responses to colorectal distension, whereas the other probiotic strains tested did not demonstrate comparable effects [50]. A separate study using a rat model of post-inflammatory colonic hypersensitivity also found that administration of this strain reduced visceromotor responses to colorectal distension [97]. These findings suggest that effects on visceral sensitivity may be strain-specific. However, both studies were conducted in animal models, and their findings cannot be directly extrapolated to gastrointestinal symptoms in humans or to adverse effects associated with semaglutide.

Clinical studies have also investigated the effects of selected *Bifidobacterium animalis* subsp. *lactis* strains on intestinal transit. In a randomized, double-blind, crossover study involving 36 healthy women, consumption of a fermented milk product containing *B. animalis* subsp. *lactis* DN-173 010 significantly shortened total and sigmoid colonic transit time compared with the control product [98]. Another randomized clinical trial investigated a fermented milk product containing DN-173 010 in individuals with constipation-predominant irritable bowel syndrome (IBS-C), reporting improvements in abdominal distension and selected gastrointestinal transit-related outcomes [45]. In addition, a 14-day randomized trial involving 100 adults with functional gastrointestinal symptoms found that supplementation with *B. animalis* subsp. *lactis* HN019 at daily doses of 1.8 × 10^9^ or 17.2 × 10^9^ colony-forming units (CFU) reduced whole-gut transit time compared with placebo [47]. However, these findings have not been consistently reproduced in subsequent trials. Two more recent randomized, placebo-controlled studies in adults with functional constipation found that HN019 was not superior to placebo in improving the primary bowel-function outcomes [77,99].

Taken together, the available evidence suggests that the effects of probiotics on visceral sensitivity and intestinal transit are strain-specific and may vary according to the study population and outcome assessed. Although selected strains have demonstrated effects in preclinical models or in clinical populations with gastrointestinal symptoms, these findings cannot be generalized to other probiotic strains or gastrointestinal outcomes. Furthermore, the available evidence does not establish that these strains prevent or alleviate semaglutide-associated gastrointestinal adverse effects. Direct clinical studies are needed to determine whether specific probiotic strains can improve gastrointestinal tolerability during GLP-1 receptor agonist treatment.

#### 3.3.6. Potential Tolerability Concerns of Probiotics and Prebiotics

Potential tolerability concerns should also be considered when evaluating probiotic and prebiotic supplementation during semaglutide therapy. Although selected probiotic strains may improve particular gastrointestinal outcomes in specific clinical settings; their effects are not universal and may vary according to the strain, dose, and individual characteristics. Fermentable prebiotics, such as fructooligosaccharides (FOS), may cause gastrointestinal symptoms, including gas production, abdominal distension, and bloating in some individuals. However, a systematic review and meta-analysis of randomized controlled trials found no significant overall differences in gastrointestinal adverse symptoms between FOS supplementation and control groups, suggesting that these effects are not consistently observed across clinical studies [100].

Patients with clinically significantly delayed gastric emptying or gastroparesis require additional consideration. Clinical guidance for gastroparesis emphasizes dietary strategies such as consuming small-particle foods to improve symptom relief and gastric emptying [101]. Dietary fiber intake may also need to be adjusted according to symptom severity and individual tolerance. Consequently, increasing fiber intake or introducing fermentable prebiotics should not be assumed to improve gastrointestinal tolerability in this population.

The type and amount of fiber should be individualized according to clinical assessment. Furthermore, although probiotics are generally well tolerated in many studied populations, rare, serious infections associated with probiotic microorganisms have been described in case reports and case series, particularly among vulnerable patients [102]. These reports do not establish the incidence of such complications but support a cautious, patient-specific approach to probiotic use in individuals with significant underlying illness or immune compromise. Overall, probiotic supplementation should be evaluated on a strain-specific and patient-specific basis. Direct clinical evidence remains insufficient to establish the safety, tolerability, or efficacy of probiotics or prebiotics specifically in patients experiencing severe gastrointestinal adverse effects during semaglutide therapy.

## 4. Discussion

### 4.1. Main Findings and Gastrointestinal Tolerability of Semaglutide

The present narrative review highlights the potential relevance of the gut microbiota to gastrointestinal tolerability and metabolic outcomes during semaglutide therapy. Semaglutide has established clinical benefits in glycemic control and weight management, supported by extensive evidence from the STEP, SUSTAIN, and PIONEER clinical trial programs [4,5,12,13,14]. By activating GLP-1 receptors, semaglutide enhances glucose-dependent insulin secretion, suppresses inappropriate glucagon secretion, and improves glycemic control. It also acts on pathways involved in appetite regulation and satiety, reducing hunger and energy intake and thereby promoting clinically meaningful weight loss [103]. Beyond its effects on glycemia and body weight, clinical trials have demonstrated cardiovascular benefits in specific high-risk populations, further supporting its clinical importance [16,104]. However, gastrointestinal adverse effects, particularly nausea, vomiting, diarrhea, and constipation, remain important clinical considerations because they may affect treatment tolerability and adherence [4,5,23]. Identifying strategies that could support gastrointestinal tolerability without compromising the established benefits of semaglutide therefore remains an important research priority. The evidence reviewed suggests that selected probiotic strains may influence particular gastrointestinal outcomes, including bowel function and intestinal transit, in clinical populations not receiving semaglutide. However, the available evidence differs according to the strain, clinical context, and symptoms assessed. Hence, the indirect rationale for investigating probiotics is currently stronger for selected lower gastrointestinal outcomes, such as constipation and diarrhea, than for upper gastrointestinal symptoms, including nausea, vomiting, and early satiety. Whether these effects translate into improved gastrointestinal tolerability during semaglutide treatment remains unknown, as direct clinical evidence is currently lacking.

Importantly, changes in gut microbial composition or function should not automatically be interpreted as clinically meaningful benefits. The available studies investigating microbiota changes during semaglutide treatment remain limited, and differences in study populations, intervention characteristics, and microbiota assessment methods restrict comparisons between findings. Although selected probiotics may have potential gastrointestinal or metabolic benefits, their effects cannot be generalized across strains or assumed to enhance the efficacy of semaglutide. Probiotics should therefore be considered a potential adjunctive strategy for further investigation rather than an established means of improving semaglutide tolerability or metabolic outcomes. Well-designed, adequately powered, strain-specific randomized controlled trials are needed to determine whether these proposed benefits translate into meaningful improvements in patients receiving semaglutide.

### 4.2. Potential Complementary Effects of Probiotics and Semaglutide

An important question arising from the available evidence is whether probiotics could complement semaglutide therapy by influencing gastrointestinal function and metabolic health. Although semaglutide directly activates GLP-1 receptors, selected probiotic strains may influence gut microbial composition and metabolic activity [80,81]. These distinct mechanisms provide a biological rationale for investigating potential complementary effects; however, the existence of shared physiological pathways does not establish an additive or synergistic clinical benefit.

One possible link is the gut–brain axis. The gut microbiota and its metabolites can communicate with the central nervous system through neural, endocrine, immune, and metabolic pathways, potentially influencing gastrointestinal motility, inflammatory signaling, and symptom perception. Microbial metabolites, particularly short-chain fatty acids (SCFAs), represent one potential link between probiotic supplementation and semaglutide therapy. SCFAs may influence intestinal barrier integrity, immune regulation, and enteroendocrine signaling [78,80]. Although SCFAs can stimulate the secretion of endogenous GLP-1 through receptor-mediated pathways [82], this physiological response differs from the direct pharmacological activation of GLP-1 receptors by semaglutide. SCFAs may enhance GLP-1RA efficacy by improving insulin sensitivity and stimulating endogenous GLP-1 secretion [83]; however, evidence supporting this possibility remains limited.

Dietary fiber may also be relevant because it provides a substrate for microbial fermentation and SCFA production [78,105]. Reduced food intake during semaglutide treatment could affect fiber intake in some patients, potentially influencing gut microbiota composition and metabolism [106,107]. Dietary fiber interventions have been shown to modify gut microbiota composition and, in some cases, alter short-chain fatty acid production [106,107]; however, whether reduced fiber intake during semaglutide therapy leads to similar changes remains unknown.

Taken together, it remains unclear whether differences in fiber intake or probiotic supplementation modify semaglutide-associated gastrointestinal tolerability or metabolic outcomes. Furthermore, the suitability of fermentable fiber or probiotic interventions may depend on individual gastrointestinal symptoms and tolerance, as discussed in Section 3.3.6.

Overall, because of limited available evidence, direct clinical trials are needed to determine whether specific probiotic strains, alone or in combination with an appropriate dietary fiber intake, provide additional benefits in gastrointestinal tolerability, and efficacy of semaglutide therapy.

### 4.3. Timing of Probiotic Supplementation

Timing of probiotic supplementation may be an important consideration in investigating its potential role in conjunction with semaglutide therapy. Changes in gut microbial composition and function are linked to obesity and type 2 diabetes, suggesting that some patients may already have an altered intestinal microbial environment prior to the start of pharmacological treatment. This opens up the possibility of probiotic supplementation prior to semaglutide therapy to support microbial homeostasis and gastrointestinal function prior to treatment.

Another possibility might be to start probiotics at the same time as semaglutide. Comparison of probiotic supplementation initiated prior to semaglutide versus at the time of treatment onset can help determine whether prior modulation of gut microbiota provides any additional benefit in gastrointestinal tolerance, adherence to treatment, or metabolic response. These studies could also determine if baseline microbiota characteristics are associated with response to either intervention.

Another important issue concerns the period following semaglutide discontinuation. Increased appetite and weight regain have been reported after discontinuation of treatment with semaglutide [3,108], illustrating the difficulty of sustaining weight loss after stopping pharmacological therapy. If the gut microbiota is involved in metabolic regulation and energy balance, a microbiota-supporting intervention after semaglutide discontinuation could be an additional approach to study. Future studies could therefore explore whether continued probiotic supplementation is beneficial for sustaining weight loss compared with withdrawal of both interventions after discontinuation of semaglutide.

Taken together, these considerations suggest that the interaction between semaglutide, microbiota, microbial metabolites, and dietary factors, particularly fiber intake, may be clinically relevant. Against this background, different probiotic treatment strategies warrant systemic evaluation. These different treatment strategies may provide important insights into whether probiotics are most useful as pre-treatment, as a concurrent supportive therapy or as a longer-term intervention after discontinuation of semaglutide. Randomized controlled trials comparing these approaches with systemic evaluation of gut microbiota composition, gastrointestinal symptoms, dietary intake, body weight, glycemic control, and adherence to treatment could help to clarify whether the timing and duration of probiotic supplementation influence clinical outcomes. Additionally, the probiotic strain or strains, dose, and route of administration should be specified through clinical trials. To date, no clinical trials have established the optimal probiotic strain, dose, duration, or timing relative to semaglutide initiation.

### 4.4. Limitations and Research Gaps

This narrative review has several strengths. It integrates findings to provide a broader conceptual framework for understanding the potential interaction between semaglutide therapy, gastrointestinal tolerability, gut microbiota, and probiotic interventions. However, several limitations must be acknowledged. As a narrative review, the literature selection was not based on a predefined systematic search strategy with explicit inclusion and exclusion criteria, which increases the risk of selection bias and limits reproducibility. No formal standardized assessment of study quality or risk of bias was performed, and quantitative synthesis was not undertaken. Consequently, conclusions should be regarded as hypothesis-generated and interpretative rather than definitive.

Hence, probiotic effects are highly strain-, dose-, formulation-, duration- and host-characteristic-dependent. As a result, one probiotic strain or combination cannot be extrapolated to other probiotic products. Differences in baseline microbiota, diet, metabolic status, and concomitant medications may also contribute to variability in response. In addition, human studies directly evaluating semaglutide-associated changes in the gut microbiota remain limited, with several available studies involving relatively small sample sizes and short follow-up periods [60,61]. Importantly, these studies investigated the effects of semaglutide on the gut microbiota but did not evaluate whether probiotic supplementation modifies semaglutide-associated gastrointestinal symptoms or microbiota changes. Thus, direct clinical evidence evaluating the efficacy and safety of probiotic supplementation specifically in patients receiving semaglutide remains very limited.

Most of the evidence discussed in this review concerns probiotics, gut microbiota, gastrointestinal function, or dietary factors independently of semaglutide treatment, while differences in sample collection and processing, DNA extraction methods, sequencing technologies, and bioinformatic pipelines can influence reported microbiota profiles and limit comparisons between studies [109,110]. These sources of heterogeneity may contribute to inconsistent findings and make it difficult to determine whether observed microbial changes translate into clinically meaningful outcomes. Therefore, the proposed complementary effects of probiotics and semaglutide are primarily based on biological plausibility and indirect evidence rather than demonstrated clinical benefits.

Finally, there is a lack of evidence on the optimal time and duration of probiotic supplementation relative to semaglutide therapy. More specifically, it is unclear if probiotic supplementation started before semaglutide, started together with the treatment, or maintained after semaglutide will result in different outcomes. This is a significant gap in future clinical research.

## 5. Conclusions

In conclusion, the current evidence suggests that probiotics improve gastrointestinal function through a variety of interconnected mechanisms, including re-establishment of microbial homeostasis, enhancement of intestinal barrier function, modulation of immune responses, regulation of intestinal motility, and production of beneficial microbial metabolites. These mechanisms may improve the health of the gastrointestinal tract and enhance the metabolic effects of semaglutide. However, while there is a sound biological rationale for the use of probiotics based on evidence from other gastrointestinal disorders, direct clinical evidence supporting probiotics in reducing semaglutide-induced gastrointestinal adverse effects is limited. More well-designed clinical trials are needed to clarify the potential role of probiotics as an adjunct to semaglutide therapy.

## Figures and Tables

**Table 1:** Effect of Probiotic Strains on Gastrointestinal Symptoms in Contexts Other Than Semaglutide Therapy.

Probiotic Strain	Population/Model	Dose	Duration	Main Findings
*Lactobacillus acidophilus* Rosell-52 + *Bifidobacterium longum* Rosell-175	Adults with stress symptoms (*n* = 75)	3 × 10^9^ CFU per sachet	3 weeks	Reduced stress-related abdominal pain and nausea/vomiting in a placebo-controlled trial
*Bifidobacterium animalis subsp. lactis* V9	Adults with advanced gastric or esophageal cancer receiving chemotherapy (*n* = 104)	2 × 10^10^ CFU/day	18 weeks	Improved quality-of-life scores for nausea and vomiting during chemotherapy
*Lactiplantibacillus plantarum* APsulloc 331261 (GTB1)	Adults with IBS-D	1 × 10^10^ CFU/day	4 weeks	Reduced *Escherichia–Shigella* abundance and improved abdominal pain, bloating, diarrhea, and global IBS symptoms
*Bifidobacterium longum* BB536	Adults with IBS-D	1 × 10^10^ CFU/day	8 weeks	Improved stool consistency and reduced abdominal pain and bloating
*Bifidobacterium longum* BB536 + *Lactobacillus rhamnosus* HN001 + vitamin B6	Adults with IBS (*n* = 25)	Combination formulation; exact CFU dose not reported in abstract	1 month per treatment period (crossover trial)	Reduced abdominal pain, bloating, and disease severity; improved colonic permeability; stool consistency improved in IBS-D subgroup
*Lactobacillus rhamnosus* GG	Adults with IBS	1.2 × 10^10^ CFU/day	6 weeks	Reduced abdominal pain and global IBS symptoms
*Lactiplantibacillus plantarum* 299v	Adults with IBS	1 × 10^10^ CFU/day	4 weeks	Improved global IBS symptoms, abdominal pain, and bloating
*Lactiplantibacillus plantarum* 299v	Adults with IBS	1 × 10^10^ CFU/day	8 weeks	NS improvement in global IBS symptoms, abdominal pain, or quality of life
*Bifidobacterium animalis* DN-173010	Adults with IBS-C	1.25 × 10^10^ CFU/day	4 weeks	Reduced abdominal distension and improved gastrointestinal transit and symptom severity
*Lacticaseibacillus rhamnosus* IDCC 3201 (RH3201)	Adults with IBS-C	Probiotic capsule; strain-specific dose	8 weeks	Improved constipation-related symptoms, bloating, and bowel satisfaction
Probiotic strain	Population/Model	Dose	Duration	Main findings
*Bifidobacterium lactis* HN019	Adults with functional GI symptoms	1.8 × 10^9^ or 17.2 × 10^9^ CFU/day	14 days	Reduced whole-gut transit time and frequency of several upper and lower GI symptoms, including constipation, abdominal pain, nausea, and flatulence
*Bifidobacterium bifidum* G9-1 (BBG9-1)	Adults with type 2 diabetes and GI symptoms	12 mg/day	12 weeks	Improved gastrointestinal symptom scores and constipation-related symptoms
*Bifidobacterium lactis* NCC2818	Adults with chronic constipation	1.5 × 10^10^ CFU/day	4 weeks	NS improvement in whole-gut transit time, stool output, GI symptoms, or quality of life
*Lactobacillus rhamnosus* GG	Adults with colorectal cancer receiving 5-FU-based chemotherapy (*n* = 150)	1–2 × 10^10^ CFU/day	24 weeks	Associated with less grade 3–4 chemotherapy-related diarrhea and abdominal discomfort; the trial also evaluated fiber supplementation
*Lactobacillus acidophilus* + *Bifidobacterium bifidum*	Patients with locally advanced cervical cancer receiving concurrent chemoradiotherapy (*n* = 63)	≥1 × 10^9^ CFU of each strain per capsule	During pelvic radiotherapy	Lower incidence of grade 2–3 diarrhea than placebo (9% vs. 45%)
Multi-strain *Lactobacillus*/*Bifidobacterium* preparation	Adults with functional constipation and bloating	Multi-strain formulation	2 weeks	NS improvement in the primary bloating outcome

Note. CFU, colony-forming units; GI, gastrointestinal; IBS, irritable bowel syndrome; IBS-D, diarrhea-predominant IBS; IBS-C, constipation-predominant IBS; 5-FU, 5-fluorouracil. None of the listed studies evaluated these interventions in patients receiving semaglutide; NS, not significant. The relevance to semaglutide-associated gastrointestinal adverse effects is therefore indirect.

**Table 2:** Potential Effect of GLP-1RA on Gut Microbiota and Metabolic/Intestinal Correlation.

Study	Model	GLP-1RA	Dose/Duration	Main Microbiota Finding	Metabolic/ Intestinal Correlates	Evidence
Chen et al., 2025 [60]	Human; T2D; *n* = 15	Semaglutide	0.25 → 0.5 → 1.0 mg/week; 12 weeks	↓ Firmicutes; ↑ Bacteroidota, Actinobacteriota and Proteobacteria; ↑ *Bifidobacterium*; ↓ *Blautia* and *Klebsiella*	↓ body weight, BMI, HbA1c, glucose and lipids; microbial changes correlated with metabolic indices	Direct human
Klemets et al., 2026 [61]	Human; T2D	Semaglutide	Baseline, 1, 3 and 12 months	NS change in overall microbial diversity; baseline microbial features were associated with subsequent treatment response	Baseline microbiome associated with HbA1c response	Direct longitudinal human
Duan et al., 2024 [63]	HFD-induced obese C57BL/6J mice	Semaglutide	HFD 10 weeks; intervention 18 days	HFD-associated ↓ *Akkermansia*, *Faecalibaculum* and *Allobaculum* and ↑ Lachnospiraceae and *Bacteroides* were partly reversed by semaglutide	Improved body weight, glucose tolerance and insulin resistance; ↑ tight-junction proteins and improved intestinal barrier	Preclinical
Mao et al., 2024 [64]	db/db mice	Semaglutide	0.22 mg/kg every 3 days; 16 weeks	↑ *Alloprevotella* and *Alistipes*; ↓ *Ligilactobacillus* and *Lactobacillus*	Improved intestinal integrity and metabolic/ NAFLD-related outcomes	Preclinical
da Silva et al., 2025 [65]	Preclinical	Semaglutide	Semaglutide intervention; 6 weeks	Changes in Bacteroidetes, *Bacteroides acidifaciens* and *Blautia coccoides*	Reduced intestinal inflammation and barrier dysfunction;	Preclinical
Study	Model	GLP-1RA	Dose/duration	Main microbiota finding	Metabolic/ intestinal correlates	Evidence
Sun et al., 2025 [66]	HFD-induced obese C57BL/6J mice	Semaglutide	100 μg/kg	Semaglutide reshaped gut microbial composition and microbial metabolic pathways	Improved body weight, fat mass, glucose and insulin sensitivity;	Preclinical/mechanistic
Wang et al., 2018 [67]	Human; T2D; *n* = 37	Liraglutide	42 days	↑ *Akkermansia*; ↓ *Sutterella* at day 42 compared with metformin	Findings discussed in relation to gut-barrier homeostasis	Direct human
Liang et al., 2024 [68]	Human; newly diagnosed T2D	Dulaglutide	1 and 48 weeks	microbiota composition; NS change at 1 week; significant compositional changes at 48 weeks	Microbiota changes associated with metabolic parameters	Direct longitudinal human

Note. T2D; type 2 diabetes; HFD; high-fat diet; HbA1c; glycated hemoglobin; NAFLD; non-alcoholic fatty liver disease; NS; not significant. ↑ indicates an increase; ↓ indicates a decrease in the respective parameter.

**Table 3:** Selected clinical studies of probiotic interventions targeting intestinal barrier function and inflammatory responses.

Study	Probiotic Intervention	Population	Main Findings	Evidence Limitation
Krumbeck et al., 2018 [90]	*Bifidobacterium adolescentis* IVS-1 or *Bifidobacterium animalis* subsp. *lactis* BB-12; 1 × 10^9^ CFU/day, alone or with galactooligosaccharides (GOS; 5 g/day); 3 weeks.	Adults with obesity; randomized, double-blind, placebo-controlled trial.	Improved selected measures of colonic permeability after an aspirin challenge. Effects differed by strain; combining either strain with GOS did not demonstrate synergistic benefit. Markers of endotoxemia did not change.	Permeability was assessed using surrogate sugar-recovery measures after aspirin challenge. Findings were strain-specific and do not establish clinical benefit during semaglutide treatment.
Choi et al., 2026 [91]	*Bifidobacterium bifidum* BGN4; 9 × 10^9^ CFU/day; 8 weeks.	Adults with excess adiposity; 60 randomized, 58 completed the trial.	Serum zonulin and TNF-α decreased, NS change in hs-CRP, BMI, and body-fat percentage. Selected gut microbial taxa also changed.	NS primary inflammatory outcome; no participants were receiving semaglutide.
Hegazy and El-Bedewy, 2010 [93]	*Lactobacillus delbrueckii* and *Lactobacillus fermentum* preparation; 1 × 10^10^ CFU/day, alongside sulfasalazine 2400 mg/day; 8 weeks.	30 patients with mild-to-moderate ulcerative colitis; 15 received probiotic plus sulfasalazine and 15 received sulfasalazine alone.	The adjunctive probiotic group showed reductions in selected inflammatory measures, including colonic IL-6, TNF-α/NF-κB p65,	Small study; concomitant sulfasalazine complicates attribution of effects to the probiotic alone. Strains were not described at strain-level resolution;
Jung et al., 2022 [94]	*Lactiplantibacillus plantarum* CJLP243; 1 × 10^10^ CFU/day; 2 months.	Patients with functional diarrhea and elevated fecal calprotectin; 24 randomized, 22 completed.	The study reported improvement in diarrhea-related symptom relief and a reduction in fecal calprotectin in the probiotic group.	Small exploratory trial with few completers; within-group changes should not be interpreted as definitive evidence of a treatment effect. The population and condition differ from semaglutide-associated symptoms.
Shin et al., 2018 [95]	*Lactobacillus gasseri* BNR17; 1 × 10^10^ CFU/day; 8 weeks.	Patients with diarrhea-predominant irritable bowel syndrome (IBS-D); 60 randomized, 51 completed.	Selected IBS symptoms and quality-of-life measures improved; colonic transit time increased in the probiotic group, with some participants shifting from fast toward more normal transit. Fecal microbiota changes were also reported.	Single-center trial with a modest sample; results concern IBS-D and cannot be extrapolated directly to semaglutide-associated gastrointestinal adverse effects.
Eghbali et al., 2023 [96]	LactoCare synbiotic; 5 × 10^9^ CFU per administration, twice daily; 7 days.	113 children with acute lymphoblastic leukemia receiving maintenance chemotherapy; 58 assigned to LactoCare and 55 to Placebo.	Compared with placebo, supplementation was associated with lower occurrence of chemotherapy-induced diarrhea and constipation during the observation period.	The accessible report does not clearly identify individual probiotic strains, limiting strain-specific interpretation. Pediatric chemotherapy-related gastrointestinal toxicity has different mechanisms from semaglutide-associated symptoms.

Note. CFU, colony-forming units; GOS, galactooligosaccharides; hs-CRP, high-sensitivity C-reactive protein; IBS-D, diarrhea-predominant irritable bowel syndrome, NS, not significant; None of the listed studies evaluated these interventions in patients receiving semaglutide; relevance to semaglutide-associated gastrointestinal adverse effects is therefore indirect.

## Data Availability

No new data were created or analyzed in this study. Data sharing is not applicable to this article.

## Abbreviations

The following abbreviations are used in this manuscript:

ENS	Enteric Nervous System
FAO	Food and Agriculture Organization
FOS	Fructooligosaccharides
GLP-1RA	Glucagon-Like Peptide-1 Receptor Agonist
GI	Gastrointestinal
GPR 41	G-Protein-Coupled Receptor 41
GPR 43	G-Protein-Coupled Receptor 43
GSRS	Gastrointestinal Symptom Rating Scale
HbA1c	Glycated Hemoglobin
SCFAs	Short-Chain Fatty Acids
T2D	Type 2 Diabetes
WHO	World Health Organization
